# It is time to learn from patients like mine

**DOI:** 10.1038/s41746-019-0091-3

**Published:** 2019-03-19

**Authors:** Saurabh Gombar, Alison Callahan, Robert Califf, Robert Harrington, Nigam H. Shah

**Affiliations:** 1 Department of Pathology, School of Medicine, Stanford University, CA Stanford, USA; 2 Department of Medicine, School of Medicine, Stanford University, CA Stanford, USA; 3 Duke University, Durham, NC USA

**Keywords:** Translational research, Health policy, Health services

## Abstract

Clinicians are often faced with situations where published treatment guidelines do not provide a clear recommendation. In such situations, evidence generated from similar patients’ data captured in electronic health records (EHRs) can aid decision making. However, challenges in generating and making such evidence available have prevented its on-demand use to inform patient care. We propose that a specialty consultation service staffed by a team of medical and informatics experts can rapidly summarize ‘what happened to patients like mine’ using data from the EHR and other health data sources. By emulating a familiar physician workflow, and keeping experts in the loop, such a service can translate physician inquiries about situations with evidence gaps into actionable reports. The demand for and benefits gained from such a consult service will naturally vary by practice type and data robustness. However, we cannot afford to miss the opportunity to use the patient data captured every day via EHR systems to close the evidence gap between available clinical guidelines and realities of clinical practice. We have begun offering such a service to physicians at our academic medical center and believe that such a service should be core offering by clinical informatics professional throughout the country. Only if we launch such efforts broadly can we systematically study the utility of learning from the record of routine clinical practice.

## Introduction

Randomized controlled trials (RCTs) are the gold standard of clinical evidence and the bedrock of evidence-based medicine. However, the cost of conducting RCTs, their narrow inclusion criteria, and their focus on only a subset of patient demographics, conditions, and treatments limits their applicability in the majority of scenarios encountered daily by clinicians.^1^ In 2011, Frankovich et al.^2^ reported a case of using electronic health records (EHRs) to guide the clinical care of a patient in the absence of RCT-based evidence, and in 2014, Longhurst et al.^3^ outlined a future in which health information systems help clinicians leverage patient data stored in the EHR at the point of care. Despite the promise of unlocking the treasure trove of EHR data to improve patient care, the state of affairs has not advanced much since 2011. The primary barriers are the methodological and operational challenges of distilling patient data into digestible clinical evidence that a physician can act on.

A common narrative in the popular press is that EHRs, combined with advanced computing and data science methods, are ready to transform healthcare. Given the prevalence of this perspective, and the increasing volume and availability of EHR data, one could imagine that it is feasible to extract knowledge with a high clinical value from EHRs in a fully automated manner with little expert input. However, much of the promise of the healthcare data revolution^4^ is hype that fails to acknowledge the complex nature of clinical decision making.^5^ A “one size fits all” solution is unlikely to work in such settings. Furthermore, medical practitioners have highlighted ethics and safety concerns^6,7^ in turning over care decisions to machine-based systems that operate over incomplete and biased EHRs^8^ without physician input. Shortliffe et al.^9^ recently highlighted the six capabilities a system must possess in order to support clinical decisions including transparency, rapid turnaround, ease of use, the relevance of answer, respect for users, and solid scientific footing.

We believe that such challenges—of getting reliable data out of the EHR and satisfying the criteria of successful clinical decision support—are best overcome via a specialty consultation service. Such a service would use state-of-the-art analytic methods to glean reliable insights out of the EHR and have medical domain expertise to contextualize results for clinical decision making. Such a service would be staffed by a team comprised of a clinical informatics trained physician for interfacing with the requesting provider and to provide clinical context when interpreting findings, an EHR data specialist to create patient cohorts, and a data scientist to perform statistical analyses. The setup as a specialty consult is radically different from the popular paradigm of self-serve AI-enabled tools that undertake data processing behind the scenes and directly present the results to a physician for interpretation. We believe that an “expert in the loop” set up is necessary to strike a balance between efficiency and rigor given the limitations of the data, and the inference methods.^10^

We launched an IRB approved pilot of such a service at our academic medical center, to study the feasibility of integrating on-demand evidence into routine patient care. We propose that such a service should be core offering by clinical informatics professionals throughout the country. For many medical centers, a significant challenge in offering such a service—beyond the staffing—is the rapid creation of patient cohorts. Depending on available tools and personnel, cohort generation may take several weeks, which is untenable for care decisions that must be made within days. To enable the consult service, we have developed a search engine that indexes patient timelines for building cohorts matching a clinical phenotype, identifying controls for comparative analyses, and searching for outcomes of interest, with sub-second response times.^11^ After cohorts are created, established analysis approaches, including propensity score matching to identify similar patients, survival analysis, and causal inference can be used to compare outcomes and provide support for clinical decisions.^10^ Upon completion of a consult, the requesting clinician receives a report which includes a summary of the cohort(s) of interest, a description of the analyses, the results, and, most importantly, a clinical interpretation to contextualize the results and explain their limitations (see an example consult request and summary of results in Box 1).

The idea of examining “patients like mine” to estimate risk and select the optimal treatment is not new—the first efforts date back to the 1970s.^12^ The informatics consult service we envision connects clinicians with researchers capable of answering different kinds of clinical questions using state-of-the-art analysis methods; removing the bottlenecks in generating and using evidence learned from the EHR. Having such a service does not eliminate the issue of data incompleteness. The importance of clinicians’ role in accurately defining patient phenotypes, and that of data scientists in empirically assessing the robustness of statistical analyses and their results, thus cannot be overemphasized.

To be sure, providing such a service incurs costs for personnel and IT infrastructure, and it is unclear if these will be reimbursable via existing payment models. However, as reimbursement models move away from fee for service models to value-based models, and given the fact that a small number of conditions result in a large fraction of healthcare costs, the economics of improving population management using such insights may be favorable. Extrapolating from current operating costs, we estimate that a service which can answer 15–20 high complexity consults a week would require an operating budget of ~$600,000 a year; which comes to about $550 per consult. This cost could be justified via improved clinical outcomes obtained for complex cases but more data must be gathered before a proper return on investment (ROI) analysis can be conducted for such a novel service.^13^ Additional considerations for the ROI include potential new reimbursement models for a “second opinion” from aggregate patient data and cost offsetting by savings from quality improvement or value-based care initiatives. Furthermore, demonstrating value to the ordering providers is a crucial piece in justifying the service’s impact. Therefore, in the current pilot, we ask participating clinicians their likelihood of recommending the service to others, as well as track repeat usage.

The demand for and benefits gained from such a consult service will naturally vary by practice type and data robustness. However, the existing gap between the evidence available in clinical guidelines and what is needed for safe, effective personalized treatment costs both resources and lives.^14^ We cannot afford to miss the opportunity to leverage the tremendous value of patient data captured every day via EMR systems to close this evidence gap. Only if we launch such efforts to bring evidence distilled from similar patients to bear on decision making at the bedside, can we systematically study the utility of learning from the record of routine clinical practice in a true learning healthcare system.^15,16^ The availability of data and effective methods to analyze it have been transformative in many other settings—it is time for healthcare to test the same potential.

Box 1 An example consult request with a summary of analysis and results produced by our service **Consult request:** Do patients who have a first-time mildly elevated kappa or lambda free light chains go on to develop malignancy?

**Question formulation:** Patients who have an incidental mildly elevated first free light chain test result are patients 18 or older, with no history of hematologic malignancy, with a first kappa free light chain test result between 2.1 and 5.0 (inclusive) or a lambda free light chain test result between 2.7 and 5.0 (inclusive), a normal free light chain ratio test result, and a normal serum protein electrophoresis (SPEP) test result (if any) within 30 days on either side of their free light chain test. Patients with a normal first free light chain test result are patients 18 or older, with no history of malignancy, with a normal free light chain ratio test result, a kappa free light chain test result between 0.3 and 2.0 (inclusive) and a lambda free light chain test result between 0.6 and 2.6 (inclusive). A normal free light chain ratio test result is between 0.3 and 1.6 (inclusive). Hematologic malignancy is defined as any of ICD9 200–209, ICD9 238.6, ICD9 238.7, ICD10 C81-C96 or ICD10 D47. The outcome of interest is time to first hematologic malignancy.

**Analysis type:** Survival analysis with right-censoring to account for differences in follow-up time among patients, and using three choices of matching algorithms to restrict analysis to similar patients.

**Results:** Our analysis identified 1012 patients whose first serum free light chains were normal, and 760 whose first serum free light chains were mildly elevated despite having a normal ratio. The cohort with mildly elevated light chains had significantly lower malignancy-free survival (p < 0.001). This finding held true when using propensity score matching to control for confounding by observable patient characteristics (age, sex, previous diagnoses, treatments, et cetera).
